# A severe case of visceral leishmaniasis and liposomal amphotericin B treatment failure in an immunosuppressed patient 15 years after exposure

**DOI:** 10.1186/s12879-017-2192-4

**Published:** 2017-01-17

**Authors:** Anna Eichenberger, Annina E. Buechi, Andreas Neumayr, Chistroph Hatz, Andri Rauch, Marc Huguenot, Eva Diamantis-Karamitopoulou, Cornelia Staehelin

**Affiliations:** 1 Department of Infectious Diseases, Bern University Hospital (Inselspital), University of Bern, PKT2B, CH-3010 Bern, Switzerland; 2 Swiss Tropical and Public Health Institute, Basel, Switzerland; 3 University of Basel, Basel, Switzerland; 4 Department of Nephrology, Bern University Hospital (Inselspital), University of Bern, Bern, Switzerland; 5 Institute of Pathology, University of Bern, Bern, Switzerland

**Keywords:** Visceral leishmaniasis, Immunosuppression, Latency, Fever in returning traveller, *L. donovani*

## Abstract

**Background:**

Visceral leishmaniasis (VL) is a protozoan disease, which is responsible for 200.000–400.000 yearly infections worldwide. If left untreated, the fatality rate can be as high as 100% within 2 years. 90% of cases occur in just six countries: India, Bangladesh, Sudan, South Sudan, Ethiopia and Brazil. It is thus a disease rarely seen by physicians in Europe or North America. We report on the fatal case of VL in an 80-year-old immunosuppressed patient who presented with a latency of over 15 years after having visited an endemic region. This is the first report showing such extreme latency of VL in a European traveller. This case is furthermore unusual because it suggests primary treatment failure to liposomal amphotericin B.

**Case presentation:**

An 80-year-old man who was on immunosuppressive treatment due to a non-specific inflammatory disease of the liver and kidney presented to our hospital with recurrent fever, fatigue and bloody diarrhoea. Histopathological analysis from a colon biopsy showed intracellular amastigotes. The diagnosis of VL was confirmed by polymerase-chain-reaction (PCR) of the colon biopsy. PCR was also performed in plasma, a bronchopulmonary lavage, a lymph node, liver and bone marrow biopsy and proved *L. donovani* as causative species. The disseminated infection was unresponsive to treatment with liposomal amphotericin B as recommended in immunosuppressed individuals despite stopping immunosuppressive treatment.

**Conclusion:**

Imported cases of VL to non-endemic regions are increasing due to extensive international travel and migration. Furthermore, the increase of elderly patients and immunosuppressed individuals, secondary to HIV, post-transplant and chemotherapeutic agents, has resulted in an increase of VL also in endemic regions of Europe. It is thus important for physicians to be able to recognize the infection. This case also demonstrates treatment failure to amphotericin B, which was only a known problem in patients with HIV until now. The knowledge of this as a possible complication is important for specialists treating the disease.

## Background

Visceral leishmaniasis (VL; also known as “kala-azar”) is a neglected parasitic disease caused by the protozoans *Leishmania donovani* and *L. infantum*. Worldwide 0,2 to 0,4 million new clinical cases are diagnosed annually, 90% of these occurring in just six countries: India, Bangladesh, Sudan, South Sudan, Ethiopia and Brazil [1, 2].

Leishmania parasites are transmitted through the bites of female sand-flies (genus *Phlebotomus* in the Old World and *Lutzomyia* in the New World) and multiply in different macrophage populations in the human host. Fever, splenomegaly, pancytopenia, consuming weight loss, and weakness are the main clinical features. VL is fatal if left untreated.

Today, the first line treatment for immunocompetent patients is liposomal amphotericin B, although various regimens are used in different endemic regions [3–5]. Regarding the treatment of immunosuppressed individuals, evidence-based treatment recommendations are currently limited to human immunodeficiency virus (HIV) positive patients. HIV-coinfection has been reported to increase the risk of treatment failure, relapse and mortality [6]. The World Health Organisation (WHO) equally recommends liposomal amphotericin B for these patients, yet with a higher cumulative dose of 40 mg/kg [5].

Here we report the fatal case of an 80-year-old immunosuppressed patient presenting with VL more than 15 years after having visited an endemic region and despite treatment with liposomal amphotericin B as recommended in immunosuppressed individuals.

## Case presentation

An 80-year-old man presented to our hospital in March 2015 with recurrent fever, fatigue and bloody diarrhoea. Clinical examination revealed a patient in sepsis with hypotension (86/64 mmHg), tachycardia (115/min), fever of 38.6 °C, bibasal crepitations, hepatosplenomegaly, and a bodyweight of 76 kg.

Laboratory examinations showed leucopenia of 3.1 G/l (reference range 3.5–10.5), normal haemoglobin and platelets, elevated liver enzymes with ASAT 60 U/l (<50) and ALAT 71 U/l (<50), and altered kidney function with creatinine 183 μmol/L (59–104) and a glomerular filtration rate of 29 ml/min (>59).

**Fig. 1:**
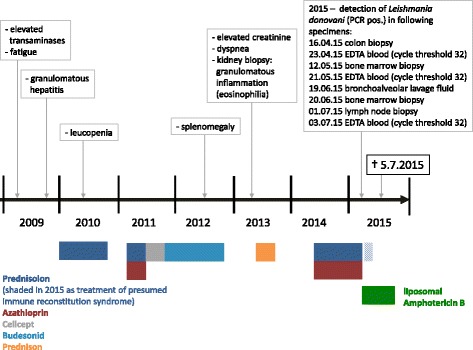
Timeline demonstrating the course of disease from first symptoms, intermittent treatment with immunosuppressive medication, to diagnosis of visceral leishmaniasis

The patient had already been evaluated for elevated liver enzymes in 2009 with a biopsy showing a non-specific granulomatous inflammation. After finding no evidence for tuberculosis and sarcoidosis he was intermittently treated for an unspecified inflammatory liver disease with high dose corticosteroids, azathioprine and mycophenolate mofetil without any apparent improvement (Fig. 1). When the kidney function deteriorated during treatment a kidney biopsy was performed in 2013. Again, histological examination showed a non-specific granulomatous inflammation. As further examinations by the nephrology department revealed no specific cause, immunosuppressive treatment with high-dose corticosteroids and azathioprine was continued and was still on-going when he presented in March 2015.

The medical history was otherwise unremarkable except for treated hypertension, diabetes mellitus type II, surgery for an abdominal aneurysm and treated Q-fever in 1984. The patient was born in Holland and moved to Switzerland as an adult. He had extensively travelled when working abroad as an engineer with longer stays till 1995 in Sudan, Bangladesh, Suriname and Indonesia. His last shorter journeys were to Indonesia and India in 2000. Since then, he had only regularly travelled to Sardinia.

**Fig. 2:**
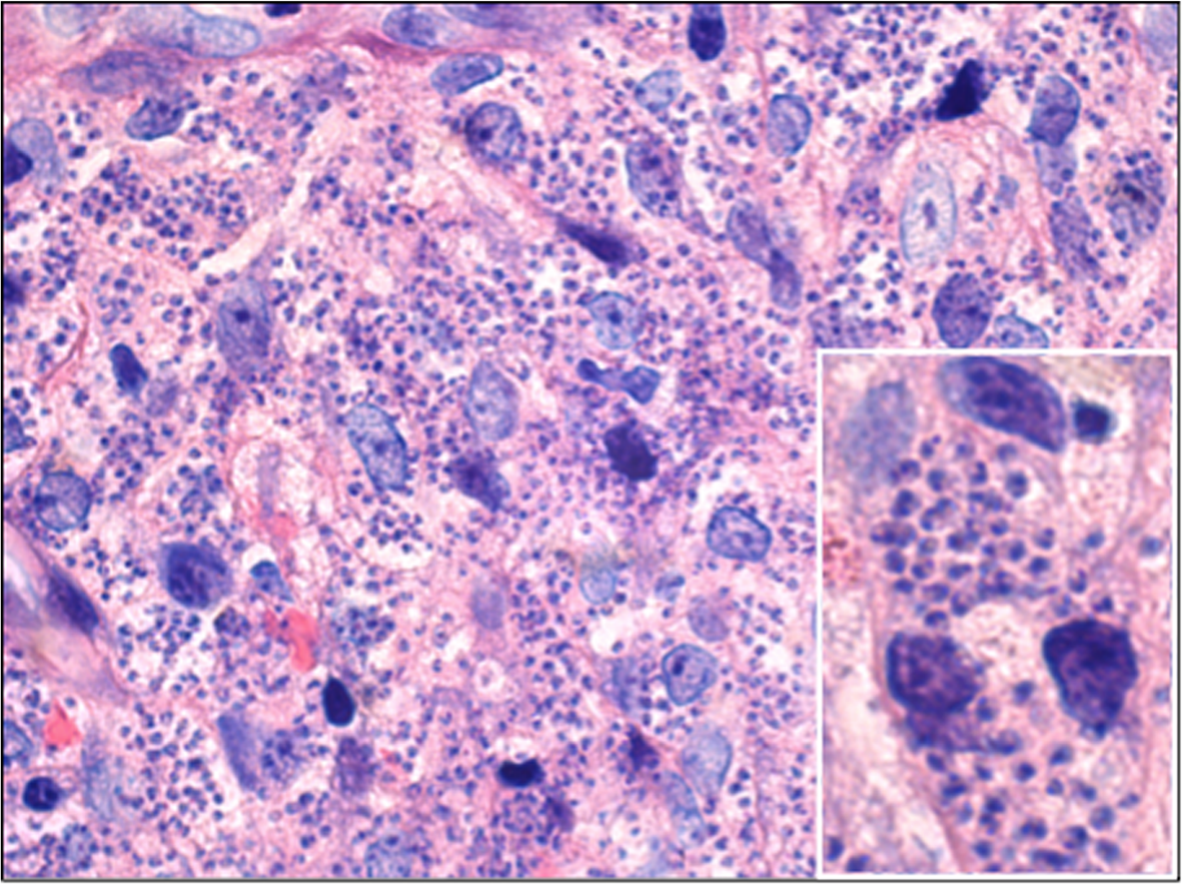
Histological preparation of colon biopsy, showing innumerable intracellular amastigotes in lamina propria macrophages (Giemsa staining, x500) and magnified section showing three enlarged macrophages (x1000)

Numerous serologic investigations were done with no pathological findings, including a negative HIV test and negative serologies for histoplasmosis and coxiella (the latter with IgG positive but IgM negative). Due to the bloody diarrhoea a colonoscopy was performed, showing extensive colitis. Histopathological analysis of the biopsy revealed intracellular amastigotes in very high density (Fig. 2). The diagnosis of VL was supported by a strongly positive serology and finally confirmed by polymerase chain reaction (PCR) from the colon biopsy as well as plasma, confirming *L. donovani* as causative species.

**Fig. 3:**
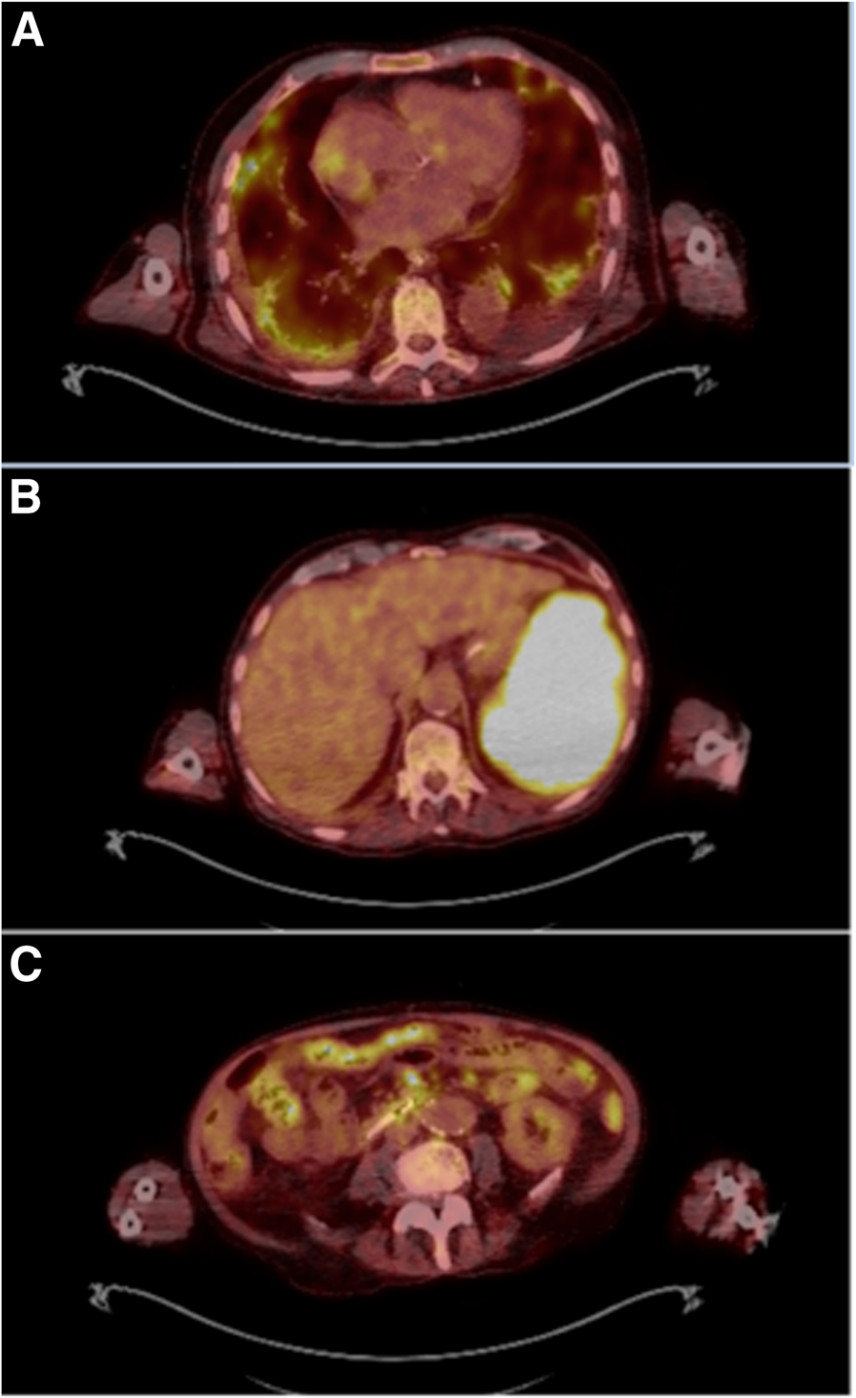
Transverse images of ^18^ F-FDG PET-CT showing intense uptake of FDG into (**a**) pleural base, (**b**) the enlarged spleen (18 cm) and (**c**) colon and peritoneal lymph nodes

Following the diagnosis, treatment with azathioprine was stopped immediately and the corticosteroids were slowly tapered. Treatment with liposomal amphotericin B was started with a dose of 3 mg/kg/day on days 1–5, 14, 21, and weekly thereafter. However, the patient’s condition continued to deteriorate with on-going fevers, wasting and progressive weakness. Further investigations were conducted to exclude any other pathology. A bronchopulmonary lavage, a lymph node biopsy, a repeated liver biopsy and two bone marrow biopsies were again PCR-positive for *L. donovani* but showed no other pathologies. A positron emission tomography–computed tomography (PET-CT) showed enhancement throughout the colon, spleen, liver, lymph nodes and basal compartments of the lungs (Fig. 3), again underlining the dissemination and severity of the disease. The patient also developed several complications during treatment, such as severe hypercalcaemia, gram-negative catheter-associated bacteremia, and an acalculous cholecystitis, though all could be controlled.

In order to monitor the treatment, real-time PCR from plasma was conducted before treatment, as well as one and two months after treatment initiation. However, the cycle threshold remained at the same value throughout the treatment course, suggesting no response to treatment. Despite receiving a cumulative dose of 40 mg/kg liposomal amphotericin B, the patient died shortly after completing treatment in July 2015, only 12 weeks after the diagnosis was made.

### Discussion

This is a case of VL in an elderly immunosuppressed patient diagnosed with an extraordinary long latency of more than 15 years after exposure. Due to the geographical distribution of *L. donovani*, the patient must have acquired the disease in either India, Sudan or Bangladesh, where he had travelled last before 2000 (India) and 1995 (Sudan and Bangladesh) [2]. In Europe *L. donovani* has recently been described only in Cyprus, however never in Sardinia [7]. This extreme latency and the fact that he only developed the classical hallmarks of the disease late into the course of his infection (hepatosplenomegaly and weight loss a few months and pancytopenia a few weeks before his death) certainly severely hampered and delayed the diagnostics (Fig. 1). In retrospect, the granulomatous inflammation, which had already been discovered in his liver biopsy in 2009 and his kidney biopsy in 2013, is well explained by VL. However, when retrospectively re-evaluating these earlier biopsies, no amastigotes were detectable. In 2015, parasitic infiltration was seen in his bone marrow, lymph nodes, colon, spleen, liver, kidneys and lung. There are case reports of VL involving unusual sites like the lung, the colon and the kidneys and VL-related cholecystitis has also been reported [8]. However, it is unusual for VL to manifest in as many organs as in our patient. This can be explained by the long-standing infection and prolonged immunosuppressive therapy.

Initially, when the treatment induced no clinical improvement and other chronic infectious diseases (such as tuberculosis, histoplasmosis or Q-fever) had been excluded after extensive diagnostic work-up, we speculated that this could be due to stopping azathioprine and tapering corticosteroids - in an immune condition similar to the immune reconstitution inflammatory syndrome (IRIS) known in acquired immunodeficiency syndrome (AIDS) patients. However, intermittent treatment with methylprednisolone did not change the clinical course. Due to the hypercalcaemia (albumin-corrected max 3,67 mmol/L), we also discussed the development of a macrophage activation syndrome, though this could not be confirmed in bone marrow biopsies.

The further differential was treatment failure due to a very high parasite burden in a patient with multiple risk factors (age, long immunosuppression and potentially several years of untreated disease). The parasite burden was certainly immense, which was shown in the histological preparation of the colon biopsy (Fig. 2) and highlighted in the PET-CT (Fig. 3).

In immunocompetent patients, treatment with liposomal amphotericin B usually leads to rapid clinical improvement and cure. Our patient failed to improve clinically, even though his cumulative dosage of liposomal amphotericin B was adjusted for immunocompromised patients and his immunosuppressive treatment had been stopped [5]. The cycle threshold of the real-time PCR remained stable for all samples. Although the real-time PCR used in our investigation is not a validated quantitative method, this finding together with his rapidly deteriorating clinical condition suggested primary treatment failure. Using an ultrasensitive real-time PCR has been shown to be a useful tool in monitoring treatment success and relapses of visceral leishmaniasis in HIV-infected patients [9]. Liposomal amphotericin B is generally the most effective and best tolerated treatment for VL. Relapses are extremely rare in immunocompetent patients, but are a known problem in HIV-positive and solid organ transplant patients [6]. Resistance to liposomal amphotericin B had not been described until a recent publication reported unresponsiveness to liposomal amphotericin B in 4 patients (1 immunocompetent, 3 immunosuppressed) [10]. Administering pentavalent antimony cured these patients.

For patients similar to ours a combination treatment could be an alternative, although there is currently not enough international data to judge the value of this approach [11]. However, studies in India have demonstrated excellent efficacy of short combination therapy and according to expert opinion, combination therapy will probably become the standard of VL treatment in the future which will allow to shorten treatment regimens, thus reducing treatment related toxicity, as well as preventing emergence of drug resistance [12]. Combination therapy may hopefully also be able to prevent relapses and failure of monotherapy in immunocompromised patients as described in this case report.

## Conclusion

International travelling has led to an increase of imported leishmaniasis (though mainly cutaneous forms) in non-endemic countries, making the recognition of this parasitic infection important, especially since many physicians are not familiar with this disease [13]. Furthermore, the increase of elderly patients and immunosuppressed individuals, secondary to HIV, post-transplant and chemotherapeutic agents, has resulted in an increase of VL in *L. infantum*-endemic regions of Europe [2, 14]. On top, unusual clinical presentations of VL may hamper the timely diagnosis in these individuals. Therefore, VL should always be included in the differential diagnosis, especially in patients with a travel history to endemic areas several decades ago and not presenting initially with the cardinal symptoms of the disease.

Patients requiring prolonged treatment with immunosuppressant drugs should undergo a detailed history of travel prior to the start of the medication, and tests investigating tuberculosis and tropical diseases such as leishmaniasis and strongyloides should be considered.

The mainstay of therapy for VL is still liposomal amphotericin B in immunocompetent patients. However, specialists have to be aware of treatment failure as a rare complication, even in immunocompetent patients, when treating the disease. Although the evidence is currently limited to a case series, salvage therapy with pentavalent antimony should be considered in patients failing to respond to amphotericin B [10].

## Abbreviations

FDG PET-CT: Fluorodeoxyglucose positron emission tomography combined with CT
HIV: Human immunodeficiency virus
PCR: Polymerase chain reaction
VL: Visceral leishmaniasis
